# Sociodemographic factors that influence COVID-19 healthcare utilization and costs in Alberta, Canada: a population-based study using the Alberta COVID-19 analytics and research database

**DOI:** 10.3389/frhs.2026.1756713

**Published:** 2026-06-24

**Authors:** Kathy Liu, Elissa Rennert-May, Zuying Zhang, Adam G. D'Souza, Alysha Crocker, Tyler Williamson, Reed Beall, Jenine Leal

**Affiliations:** 1 Department of Community Health Sciences, University of Calgary, Calgary, AB, Canada; 2 Department of Medicine, University of Calgary, Calgary, AB, Canada; 3 Department of Microbiology, Immunology, and Infectious Diseases, University of Calgary, Calgary, AB, Canada; 4 O’Brien Institute for Public Health, University of Calgary, Calgary, AB, Canada; 5 Snyder Institute for Chronic Diseases, University of Calgary, Calgary, AB, Canada; 6 Centre for Health Informatics, University of Calgary, Calgary, AB, Canada; 7 Provincial Research Data Services, Alberta Health Services, Calgary, AB, Canada; 8 Department of Surveillance and Reporting, Alberta Health Services, Calgary, AB, Canada; 9 Infection Prevention and Control, Alberta Health Services, Calgary, AB, Canada

**Keywords:** COVID-19, health equity, healthcare utilization, pandemic response, sociodemographic determinants of health

## Abstract

**Introduction:**

Understanding how sociodemographic factors influence healthcare utilization (HCU) and costs is important for equalizing care for underserved patients. This study examines how age, sex, and socioeconomic deprivation influence HCU and healthcare costs during COVID-19.

**Method:**

We completed a population-based study of adult patients with laboratory-confirmed COVID-19 diagnosis in Alberta, Canada between March 1, 2020 and December 15, 2021. Patients were stratified using standardized matrices for economic and social deprivation. Each group's healthcare utilization (both hospital- and community-based) were compared.

**Results:**

Older age and male gender are associated with significantly higher total healthcare costs. Above 50 years, number of ED visits decreased significantly (aRR = 0.78, *p* < 0.01). Social and material deprivation was associated with higher total costs and costs for ED and inpatient visits and significantly greater risk of requiring ED visits. Material deprivation was associated with shorter duration until first hospitalization. Social deprivation exerted greater effect on outpatient resources. Material deprivation had a greater effect on inpatient resources consumption.

**Discussion:**

Pandemics differentially affect patients from different social and economic backgrounds. Future studies should study the health implications associated with these differential sociodemographic factors.

## Introduction

The COVID-19 pandemic resulted in the largest increase in healthcare spending in Canada over the past 30 years, with an increase of 12% from 2019 to 2020. (1) Globally, the cost for combatting COVID-19 is estimated to be roughly $13 trillion; the exceptional financial toll has stimulated growing interest in examining how various societal determinants can impact resource utilization and financial spending in an effort to optimize current approaches to infection containment and management (2, 3).

Throughout the pandemic, patients' sociodemographic circumstances have influenced their health-seeking behaviors, clinical outcomes, and coping mechanisms (4). In many instances, this significantly exacerbated inequalities as more socially deprived individuals shouldered a disproportionately higher burden of contracting COVID-19, experiencing severe symptoms and higher mortality rates (4). Previous research mostly focused on the impact of unbalanced socio-spatial and economic determinants of health during COVID-19, tracking how this variability contributes to medical outcomes (4–9). The outcomes that were studied included robustness of long-term care services, testing rates, spread of infection, burden of comorbid diseases, mortality, alteration in social activities, rates of hospitalization, and ICU admission (4, 5, 8–11). These studies highlighted that older age, income, unemployment rate, living in deprived neighborhoods, and area-level indicators are all important factors (4, 8, 9, 12–14).

Currently, there is minimal data that clarifies how socioeconomic deprivation influences healthcare utilization (HCU), and consequent healthcare costs. This study assesses how age, sex, and social and material deprivation affect outpatient- and hospital-based COVID-19 healthcare utilization and costs in Alberta, Canada.

## Methods

This study was a population-based descriptive study using secondary data gathered across multiple healthcare facilities in Alberta, Canada.

### Patient population

This study included adult patients (18+ years) who tested positive for COVID-19 infection from March 1, 2020 and December 15, 2021. During this period, Alberta's total population was approximately 4.4 million with a 5-year population change of 4.8% (15).

First incident cases of COVID-19 were included. Patients must have received a positive nucleic acid amplification test (NAAT) on at least two specific genomic targets or one positive target with the nucleic acid sequencing at a provincial-level public health laboratory. Patients who had a confirmed positive COVID-19 test result via the National Microbiology Laboratory were also included.

Wave 1 occurred between March to June 2020; wave 2 between July 2020 and February 2021; wave 3 between March 2021 to June 2021; and wave 4 between July 2021 to December 2021 (16, 17).

### Data sources

Preliminary data was gathered through the Alberta COVID-19 Analytics and Research Database (ACARD). This is a province-wide database that was developed with Alberta Health Services' Data and Analytics Department and the Centre for Health Informatics at the University of Calgary. ACARD includes public health, clinical, laboratory, pharmacy, administrative, and financial data. Patients’ personal health numbers (PHNs), birth dates, and sex were used to link data together from the following data sources:

1. *Alberta Precision Laboratories (APL):* All laboratory-confirmed positive COVID-19 test results that were reported to APL were included. The Public Health Laboratory (ProvLab) is operated by APL and gathers COVID-19 lab test results in Alberta.
2. *AHS Enterprise Data Warehouse (EDW):* This study incorporated information from the Discharge Abstract Database (DAD), the National Ambulatory Care Reporting System (NACRS), Vital Statistics, the Alberta Continuing Care Information System (ACCIS), Practitioner Claims, the Paris and Meditech medical information systems and the Pharmaceutical Information Network (PIN) (Appendix: Table 1).

### Patient demographics and definitions

Patients were followed for six months after their positive COVID-19 test. To ensure accuracy, patient demographics were collected from all sources. Data included age deciles, sex, Elixhauser Co-morbidity Index, long-term care status, location of test collection, and infection symptoms (18).

Socioeconomic status was reported according to the Pampalon deprivation index that includes material deprivation index (based on education, employment status, and average income) and social deprivation index (based on the proportion of individuals over the age of 15 years who are living alone; who are separated, widowed, or divorced; and proportion of single-parent families) (19). Higher quintiles represent greater deprivation (19). The individual components of each index are derived from Canadian census data at the dissemination area level that is linkable to postal code and presented based on quintiles. Elixhauser co-morbidities were defined using the *International Statistical Classification of Diseases and Related Health Problems, Tenth Revision, Canada (ICD-10-CA)* diagnosis codes from DAD and NACRS and ICD-9 diagnosis codes from Practitioner Claims in the 2 years prior to the COVID-19 collection date (18).

New outpatient medications, obtained from PIN, included all medication dispensations that occurred one day before and up to 183 days after a patient's first positive COVID-19 test. New prescriptions were defined as the first instance of the prescription. Dispensations were combined if the same drug had a start date within 3 days of the expected end date of the previous prescription. PIN includes medications dispensed in the community, but not in hospitals. Dispensations captured by PIN are those that patients pay for out-of-pocket or through insurance, which has a variety of co-payment levels.

Inpatient and outpatient costs were determined by multiplying resource intensity weights in DAD and NACRs by the cost of a standard hospital stay from the Canadian Institute for Health Information (20, 21). Prescription costs were derived from the Alberta Drug Benefits List, which contains all medications covered under Alberta's government-sponsored supplemental health benefit programs (22).

### Outcomes

Outcomes were grouped as community- vs. hospital-based HCU and resulting costs. We stratified these outcomes by age, sex, and social and material deprivation.

- Community-based/outpatient outcomes: Emergency department (ED) and outpatient (OP) visits and new prescriptions
- Hospital-based outcomes: inpatient visits, number of days from diagnosis to first hospital admission, inpatient length-of-stay (LOS) for first admission, number of days until first ICU, number of ICU admissions

### Statistical analyses

Descriptive statistics were reported. Frequencies were used for categorical variables; means with standard deviations (SD) were calculated for normally distributed continuous variables. Where relevant, median values with interquartile ranges (IQR) were reported for skewed continuous variables.

We completed univariate and multivariate analysis to summarize healthcare utilization and costs by age, sex, and deprivation indices. Total mean costs included inpatient, outpatient, and ED visits. Community-based family physician visits and costs were not available at the time of data collection.

Generalized linear models (GLM) with a Gamma distribution and log link were used to analyze cost outcomes. Relative ratios and 95% confidence intervals (95% CI) were reported and average marginal costs were calculated using “R Function Margins: Marginal Effects Estimation” (23). Poisson regression models were used to analyze the number of hospital admissions, LOS, and number of outpatient visits given that these parameters included count data. Records with missing sex and Pampalon indices were excluded from the multivariate analyses. All statistical analyses were performed in R (4.1.0.) (24). *P*-values <0.05 were considered significant.

### Ethics and data availability

Ethics approval was obtained from the University of Calgary's Health Research Ethics Board (*REB20-0688*) and included a waiver of consent. Data used in this study is not publicly available as it comes from AHS. However, it may be obtained through ethics application to AHS.

## Results

Between March 1, 2020 and December 15, 2021, there were 255,037 adult patients who received a laboratory-confirmed COVID-19 test result in Alberta (Table 1). Most patients tested positive in the community (90.7%) and patients did not require hospitalization (91.9%). The top three most common comorbidities were diabetes (uncomplicated), hypertension, and fluid/electrolyte disorders. While analyzing comorbidities, “no contact” refers to patients who lack records to assess comorbidities. After excluding those with incomplete data, 227,374 patients (89.2% of the cohort) were included in the multivariate analyses. In Alberta, during the years covered in this study, the provincial population distribution included 19% between 0 and 14 years, 66.2% between 15 and 64 years, and 14.8% over 65 years (15). The mean age was 39 years (15).

**Table 1:** Patient characteristics.

Variable	All Patients (*N*)	Location of patient at time of test			
		Community (*N*)	ED (*N*)	Hospital (*N*)	LTC (*N*)
Total number	255,037	231,270 (90.68%)	16,831 (6.60%)	2,294 (0.90%)	4,642 (1.82%)
Sex (Female)	128,607	116,239 (90.38%)	8,423 (6.55%)	1,066 (0.83%)	2,879 (2.24%)
Age group (years)					
18–29	66,668	64,010 (96.01%)	2,445 (3.67%)	210 (0.31%)	3 (0.00%)
30–39	62,176	58,918 (94.76%)	3,015 (5.73%)	225 (0.36%)	18 (0.03%)
40–49	50,291	47,186 (93.83%)	2,883 (5.73%)	182 (0.36%)	40 (0.08%)
50–59	35,728	32,476 (90.90%)	2,860 (8.00%)	237 (0.66%)	155 (0.43%)
60–69	21,717	18,428 (84.86%)	2,481 (11.42%)	372 (1.71%)	436 (2.01%)
70–79	9,815	6,663 (67.89%)	1,842 (18.77%)	435 (4.43%)	875 (8.91%)
80+	8,642	3,589 (41.53%)	1,305 (15.10%)	633 (7.32%)	3,115 (36.04%)
Comorbidities					
AIDS	64	30 (46.88%)	22 (34.38%)	7 (10.94%)	5 (7.81%)
Alcohol	4,976	3,721 (74.78%)	896 (18.01%)	254 (5.10%)	105 (2.11%)
Blood loss anemia	295	178 (60.34%)	62 (21.02%)	23 (7.80%)	32 (10.85%)
Cardiac arrhythmias	4,133	2,475 (59.88%)	831 (20.11%)	301 (7.28%)	526 (12.73%)
Chronic pulmonary disorders	5,549	3,511 (63.27%)	1,404 (25.30%)	259 (4.67%)	375 (6.76%)
Coagulopathy	730	430 (58.90%)	151 (20.68%)	76 (10.41%)	73 (10.00%)
Congestive heart failure	2,067	904 (43.73%)	528 (25.54%)	256 (12.39%)	379 (18.34%)
Deficient anemia	1,668	1,084 (64.99%)	318 (19.06%)	115 (6.89%)	151 (9.05%)
Depression	4,378	3,315 (75.72%)	631 (14.41%)	185 (4.23%)	247 (5.64%)
Diabetes, complicated	5,269	2,805 (53.24%)	1,267 (24.05%)	488 (9.26%)	709 (13.46%)
Diabetes, uncomplicated	10,179	6,692 (65.74%)	2,341 (23.00%)	525 (5.16%)	621 (6.10%)
Drug abuse	4,287	3,185 (74.29%)	847 (19.76%)	220 (5.13%)	35 (0.82%)
Fluid/Electrolyte disorders	6,064	3,039 (50.12%)	1,702 (28.07%)	579 (9.55%)	744 (12.27%)
Hypertension, complicated	100	56 (56.00%)	24 (24.00%)	10 (10.00%)	10 (10.00%)
Hypertension, uncomplicated	8,320	5,255 (63.16%)	1,581 (19.00%)	535 (6.43%)	949 (11.41%)
Hypothyroidism	480	278 (57.92%)	80 (16.67%)	42 (8.75%)	80 (16.67%)
Liver disease	1,710	1,186 (69.36%)	337 (19.71%)	125 (7.31%)	62 (3.63%)
Lymphoma	261	162 (62.07%)	69 (26.44%)	16 (6.13%)	14 (5.36%)
Metastatic cancer	677	416 (61.45%)	128 (18.91%)	90 (13.29%)	43 (6.35%)
Obesity	1,487	1,187 (79.83%)	199 (13.38%)	56 (3.77%)	45 (3.03%)
Other neurological disorders	2,733	1,726 (63.15%)	408 (14.93%)	189 (6.92%)	410 (15.055%)
Paralysis	400	182 (45.50%)	43 (10.75%)	44 (11.00%)	131 (32.75%)
Peptic ulcer disease	605	444 (73.39%)	105 (17.36%)	30 (4.96%)	26 (4.30%)
Peripheral vascular disorders	722	408 (56.51%)	149 (20.64%)	81 (11.22%)	84 (11.63%)
Psychoses	1,279	829 (64.82%)	197 (15.40%)	127 (9.93%)	126 (9.85%)
Pulmonary circulation disorders	1,013	597 (58.93%)	249 (24.58%)	85 (8.39%)	82 (8.09%)
Renal failure	1,359	682 (50.18%)	320 (23.55%)	161 (11.85%)	196 (14.42%)
Rheumatoid arthritis	1,115	856 (76.77%)	166 (14.89%)	46 (4.13%)	47 (4.22%)
Solid tumor	2,203	1,551 (70.40%)	335 (15.21%)	183 (8.31%)	134 (6.08%)
Valvular disease	611	410 (67.10%)	108 (17.68%)	46 (7.53%)	47 (7.69%)
Weight loss	1,122	366 (32.62%)	189 (16.84%)	200 (17.83%)	367 (32.71%)
# of comorbidities					
0	102,954	91,863 (89.23%)	9,595 (9.32%)	512 (0.50%)	984 (0.96%)
1+	40,275	28,836 (71.60%)	7,228 (17.95%)	1,747 (4.34%)	2,464 (6.12%)
No contacts	111,808	110,571 (98.89%)	8 (0.01%)	35 (0.03%)	1,194 (1.07%)
Symptoms					
COVID-19 core^a^	132,691	118,380 (89.21%)	10,999 (8.29%)	1,252 (0.94%)	2,060 (1.55%)
COVID-19 expanded^b^	98,699	89,251 (90.43%)	8,338 (8.45%)	604 (0.61%)	506 (0.51%)
COVID-19 GI^c^	33,920	28,308 (83.46%)	4,799 (14.15%)	405 (1.19%)	408 (1.20%)
Other	13,683	11,034 (80.64%)	2,222 (16.24%)	255 (1.86%)	172 (1.26%)
No symptoms	114,450	106,444 (93.00%)	4,971 (4.34%)	847 (0.74%)	2,188 (1.91%)
LTC resident					
Yes	4,809	0 (0.00%)	88 (1.83%)	79 (1.64%)	4,642 (96.53%)
Pampalon social deprivation index quintile					
0	27,603	23,382 (84.71%)	1,563 (5.66%)	341 (1.24%)	2,317 (8.39%)
1	40,875	38,150 (93.33%)	2,219 (5.43%)	266 (0.65%)	240 (0.59%)
2	38,695	36,008 (93.06%)	2,080 (5.38%)	238 (0.62%)	369 (0.95%)
3	45,579	41,845 (91.81%)	3,046 (6.68%)	354 (0.78%)	334 (0.73%)
4	48,256	43,532 (90.21%)	3,490 (7.23%)	453 (0.94%)	781 (1.62%)
5	54,029	48,353 (89.49%)	4,433 (8.20%)	642 (1.19%)	601 (1.11%)
Pampalon material deprivation index quintile					
0	27,603	23,382 (84.71%)	1,563 (5.66%)	341 (1.24%)	2,317 (8.39%)
1	37,441	34,845 (93.07%)	1,790 (5.66%)	272 (1.24%)	534 (8.39%)
2	40,027	37,076 (92.63%)	2,274 (5.68%)	305 (0.76%)	372 (0.93%)
3	42,649	39,192 (91.89%)	2,671 (6.26%)	333 (078%)	453 (1.06%)
4	47,346	42,949 (90.71%)	3,566 (7.53%)	428 (0.90%)	403 (0.85%)
5	59,971	53,826 (89.75%)	4,967 (8.28%)	615 (1.03%)	563 (0.94%)

a Core symptoms: Feverish/chills (temperature not taken), conjunctival injection, conjunctivitis, cough, fever, loss of taste/smell, nasal congestion, rhinorrhea, shortness of breath/difficulty breathing, sneezing, sore throat, tachypnea.

b Expanded symptoms: Arthralgia, chest pain, encephalitis, headache, malaise, myalgia, pain (muscular, chest, abdominal, joint, etc.).

c GI symptoms: Anorexia, decreased appetite, diarrhea, nausea, vomiting.

d Other symptoms: Altered mental state, dizziness, irritability/confusion, pharyngeal exudate, prostration, seizures, other.

### Age (Table 2 and Appendix Table 2)

Compared with the youngest age group (18–29 years), increasing age was associated with significantly higher total healthcare costs (aRR = 8.65, *p* < 0.01 for age 80 years+). Older patients recorded higher marginal costs for ED, OP, and inpatient services. Patients aged 50 years and older recorded a significantly lower risk of ED visits (*p* < 0.01), with those over the age of 80 years having the lowest risk of requiring ED visits (aRR: 0.78; 95% CI 0.75–0.81; *p* < 0.01). Cost for ED visits continued to increase significantly among older patients.

**Table 2:** Community- and hospital-based healthcare utilization costs by age and sex (adjusted RRs^a^).

Age	Total costs	Cost of ED/UC visits	Cost of other OP visits	Cost of in-patient admits
aRR				
30–39	1.28 (1.15–1.43), <0.01	1.12 (1.09–1.15), <0.01	1.03 (0.83–1.27), 0.80	1.04 (0.92–1.17), 0.54
40–49	1.96 (1.74–2.21), <0.01	1.17 (1.14–1.20), <0.01	1.10 (0.87–1.38), 0.42	1.95 (1.72–2.21), <0.01
50–59	3.67 (3.25–4.16), <0.01	1.27 (1.24–1.31), <0.01	1.23 (0.96–1.59), 0.10	2.49 (2.21–2.81), <0.01
60–69	5.79 (5.12–6.55), <0.01	1.47 (1.43–1.51), <0.01	1.37 (1.05–1.79), 0.02	2.76 (2.45–3.11), <0.01
70–79	9.05 (7.96–10.3), <0.01	1.55 (1.50–1.60), <0.01	1.38 (1.00–1.90), 0.05	2.64 (2.33–2.98), <0.01
80+	8.65 (7.33–10.22), <0.01	1.55 (1.49–1.61), <0.01	1.55 (1.12–2.15), 0.01	1.93 (1.71–2.18), <0.01
Sex difference ^a^females compared to males	1.24 (1.16–1.33), <0.01	1.04 (1.02–1.05), <0.01	0.99 (0.86–1.14), 0.90	1.45 (1.37–1.53), <0.01
Marginal cost				
30–39	239.08 (141.05–337.11)	81.27 (63.79–98.75)	12.82 (−92.56–118.21)	545.67 (−560.96–1,652.30)
40–49	804.51 (646.34–962.68)	117.37 (101.16–133.59)	44.86 (−53.16–142.89)	12,806.67 (10,895.67–14,717.68)
50–59	2,244.58 (1,969.39–2,519.78)	185.84 (162.5–209.18)	105.99 (−4.18–216.16)	19,884.69 (17,962.50–21,806.87)
60–69	4,023.44 (3,390.79–4,656.08)	318.44 (291.44–345.43)	168.86 (32.22–305.49)	23,271.01 (20,783.03–25,759.00)
70–79	6,760.65 (5,461.43–8,059.87)	374.35 (344.11–404.58)	169.47 (−28.69–367.63)	21,219.92 (18,929.07–23,510.77)
80+	6,425.32 (4,804.20–8,046.45)	373.63 (334.30–412.96)	249.96 (−8.26–508.19)	11,581.46 (9,984.50–13,178.42)
Sex difference (Males)	546.55 (354.57–738.53)	31.22 (18.57–53.87)	−4.82 (−84.35–74.72)	9,571.84 (8,383.56–10,760.12)

a Variables included in multivariate analysis: age, sex, LTC resident status, number of hospital admits in the previous year, Pampalon Social and Material Deprivation Indexes, number of Elixhauser comorbidities, and presence or absence of specific comorbidities.

Costs for inpatient services continued to increase until 60–69 years, after which this decreased. Older patients recorded significantly shorter duration between COVID-19 diagnosis and first hospitalization as well as first ICU admit; longer inpatient LOS for first admit; and fewer ICU admits. As age increased, both the risk for hospitalization and total marginal costs increased significantly.

### Sex (Table 2 and Appendix Table 2)

Male patients recorded higher total marginal costs and marginal costs from ED and inpatient services. Male patients recorded significantly more hospital admits; longer LOS; shorter span between COVID-19 diagnosis and first hospitalization.

### Social deprivation (Table 3 and Appendix Table 3)

Patients in quintiles two-five of the social deprivation indices recorded significantly more ED visits compared to patients in quintile one (aRR: 0.97, 1.04, 1.04, and 1.08; *p* = 0.04, 0.01, 0.01, and <0.01). Likewise, these patients also recorded significantly higher cost for ED visits (marginal cost: $40.39; 95% CI: $22.35–58.43; aRR: 1.05, *p* < 0.01). As social deprivation increased from quintile one to four, the number of other OP visits decreased significantly (aRR: 0.56, 0.59, and 0.59; *p* < 0.01).

**Table 3:** Community-based and hospital-based costs by Deprivation Index (Adjusted RRs^a^).

Deprivation index	Quintile	Total cost	Cost of ED/UC visits	Cost of other OP visits	Cost of in-patient admits
aRR					
Social	2	0.91 (0.80–1.05), 0.20	0.97 (00.95–1.00), 0.02	0.65 (0.52–0.81), <0.01	0.95 (0.87–1.05), 0.33
	3	1.04 (0.91–1.19), 0.59	1.00 (0.98–1.03), 0.78	0.72 (0.60–0.87), <0.01	1.05 (0.96–1.14), 0.30
	4	1.02 (0.90–1.16), 0.777	1.00 (0.97–1.02), 0.88	0.61 (0.46–0.82), <0.01	1.04 (0.96–1.13), 0.34
	5	1.02 (0.90–1.16), 0.728	1.05 (1.02–1.07), <0.01	0.93 (0.73–1.19), 0.58	1.01 (0.93–1.09), 0.88
Material	2	1.12 (1.01–1.23), 0.032	1.01 (0.98–1.04), 0.43	1.00 (0.69–1.46), 1.00	1.02 (0.94–1.12). 0.60
	3	1.24 (1.12–1.37), <0.01	0.99 (0.96–1.02), 0.39	1.13 (0.79–1.61), 0.50	1.12 (1.03–1.22), 0.01
	4	1.20 (1.09–1.32), <0.01	0.98 (0.96–1.01). 0.22	0.90 (0.63–1.28), 0.56	1.07 (0.98–1.16), 0.11
	5	1.48 (1.32–1.65), <0.01	1.01 (0.98–1.03), 0.65	0.91 (0.64–1.29), 0.61	1.13 (1.04–1.23), <0.01
Marginal cost					
Social	2	−214.79 (−559.66–130.08)	−24.19 (−43.79–(−)4.6)	−222.94 (−378.79–(−)67.09)	−1,189.29 (−3,180.38–801.79)
	3	95.81 (−213.87–405.49)	2.99 (−17.17–23.14)	−176.63 (−302.02–(−)51.24)	1,231.28 (−774.96–3,237.51)
	4	47.07 (−229.86–324.01)	−1.5 (−19.28–16.28)	−246.05 (−395.95–(−)96.14)	1,068.16 (−726.38–2,862.70)
	5	58.14 (−255.21–371.50)	40.39 (22.35–58.43)	−42.00 (−194.77–110.78)	160.11 (−1,794.16–2,114.37)
Material	2	236.93 (−0.29–474.16)	9.31 (−13.11–31.74)	0.57 (−242.15–243.29)	574.01 (−1,394.04–2,542.07)
	3	486.28 (229.17–743.40)	−9.68 (−27.7–8.34)	73.47 (−127.29–274.24)	2,913.11 (811.80–5,014.43)
	4	410.64 (192.14–629.14)	−13.46 (−37.27–10.36)	−57.67 (−218.43–103.10)	1,712.91 (−132.57–3,558.38)
	5	972.05 (722.03–1,222.07)	4.75 (−14.39–23.89)	−49.76 (−233.92–134.40)	3,123.09 (1,312.09–4,934.10)

a Variables included in multivariate analysis: age, sex, LTC resident status, number of hospital admits in the previous year, Pampalon Social and Material Deprivation Indexes, number of Elixhauser comorbidities, and presence or absence of specific comorbidities.

### Material deprivation (Table 3 and Appendix Table 3)

As material deprivation increased, total marginal cost and inpatient services increased (marginal cost: $972.05 and $3,123.09, respectively. Patients who were most materially deprived demonstrated a significantly shorter duration between positive COVID-19 diagnosis until first hospital admission (aRR = 0.90, 0.84–0.97, *p* < 0.01 Appendix Table 3).

## Discussion

We completed a population-based study to evaluate the impact of sociodemographic factors on HCU and healthcare costs among incident adult cases of COVID-19 in Alberta, Canada between March 1, 2020 and December 15, 2021. Our factors of interest were age, sex, and material and social deprivation. Deprivation broadly refers to relative disadvantage observed amongst a community or group (19). Material deprivation refers to material goods or commodities, where deprivation may suggest financial scarcity (19). In comparison, social deprivation refers to the adequacy and quality of social connections or relationships, including those forged within families, workplace, and community (19). Our results suggest that increasing age and male sex are factors that incurred significantly higher total marginal costs for inpatient treatment as well as costs associated with ED and community-based OP visits. [insert main take-away from deprivation index results that will be discussed later].

As age increased beyond 50 years, the risk of requiring ED visits significantly dropped while costs continued to increase. This may suggest that older individuals are less likely to access community-based acute care services, like the ED, during the pandemic, but when they do, the cost of care is nonetheless high. Surme et al. found that Covid-19 Fear Scores were significantly higher among patients over the age of 57 years (25). It is important to consider barriers to accessing ED services for vulnerable patients. Factors such as poor baseline health status and mental illnesses cause delays in accessing acute care (26). In Italy, the prevalence of delays in care were significantly greater among patients aged 75 years and older and that individuals with co-morbidities experienced poorer access to ED services (26). Likewise, in Singapore, overall HCU decreased among elderly patients (27).

Our group previously demonstrated that severe infections requiring hospitalization and ICU are more expensive than non-hospitalized and non-ICU hospital admissions (28). This result is largely in keeping with most economics studies that have shown that HCU costs during the pandemic are largely driven by inpatient and particularly ICU treatment (29–31). In this study, we add to this and found that social deprivation is associated with greater cost for OP services, but we did not observe this same trend for material deprivation, which was associated with greater cost for inpatient services. Social support is associated with delay in seeking medical care (32, 33). Adult patients with congenital heart defect living in the more socially deprived neighborhoods are more likely to have fewer outpatient healthcare appointments but more visits to ERs, suggesting that social deprivation may lead to decreased engagement in outpatient care and in turn leads them to use more acute medical services (34).

Social deprivation includes measuring proportion of individuals who live alone or in single-parent homes. These individuals may lack social support or motivation to seek hospital-based medical help for their illness, which in turn may prompt them to rely more heavily on community-based resources. From 2004 to 2014, in Edmonton, Alberta, ED visits for influenza-like illnesses was 1.5 times higher among patients from most deprived social quintiles (35). Across Alberta, Eagles et al. found that living in deprived neighborhoods was associated with lower likelihood for being treated with endovascular thrombectomy (36). A British study found that among socially deprived patients, there was greater attendance to minor injury treatment centres vs. EDs (37). Another hypothesis for this finding may be that populations that are socially deprived may be linked to geographic remoteness, which made it even more difficult for these patients to access EDs that may be far from where they live. Moreover, individuals who have greater social deprivation may experience poorer mental health which may also discourage them from presenting to EDs (38). The exact mechanisms causing these results remains unclear and need to be further explored. Future studies can study interventions that cultivate social engagement and promote health literacy among socially deprived groups.

Previous studies show that COVID-19 had higher incidence in more deprived areas, which also have higher rates of hospitalization (6, 7, 39–43). The cause for this is likely complex and multifactorial. Healthcare delivery during the pandemic faced unprecedented challenges, particularly in Alberta where geographic spread was a major challenge for delivering timely care to all patients (44). Profession, baseline health, and health literacy may also impact healthcare seeking behavior among patients from different deprivation quintiles (45, 46). A narrative study in New Zealand highlighted preference for in-person care, long wait periods, language barriers, and poor support for attending appointments as key sources of patient dissatisfaction during the pandemic for marginalized populations (47). A Polish study found that long wait times and closure of healthcare centres were the two most common barriers encountered by patients; moreover, lack of higher education and having at least one chronic illness were factors associated with higher odds of encountering barriers to accessing healthcare (48). In Canada, Etowa et al. found that minority and immigrant patients are significantly more likely to report difficulties using emergency services/urgent care during the pandemic (49).

Our study was population-based, including patients from across Alberta. This allowed us to consult multiple data sources. Therefore, our results have wide geographic coverage to ensure that patients from different settings across the province are represented. A limitation for our study is that vaccination data was not available. This could have impacted disease severity and clinical course among the patient population. In addition, we did not examine differences in COVID-19 testing over time. This would have been an important factor to consider as different tests vary in sensitivity and specificity; they may only detect positive cases at different timepoints in the disease history, which in turn may impact hospital-based HCU and time-to-hospitalization. Also, we did not report the specific causes for HCU outcomes examined in our analysis. These may include employment status, geographic location (ex. urban vs. rural location of residence), and access to a family physician.

## Conclusion

Our population-based study featured a province-wide analysis of sociodemographic factors that influence HCU and healthcare costs among COVID-19 patients in Alberta, Canada. Our results clarify how different levels of deprivation and patient characteristics affect patterns of health services use in the community and hospitals. In the future, more studies can be replicated on a national level to better facilitate health services planning and resource allocation in all of Canada. Suggest ending with one of the policy recommendations or policy relevant observations like the utilization patterns of those from more deprived areas accessing more emergency services rather than routine community-based care.

Our findings highlight the impact of socioeconomic deprivation on health services consumption patterns during the COVID-19 pandemic. The health services consumption patterns underscore the strong reliance for more deprived patients to access emergency services and less utilization of other routine community-based medical care during COVID-19. Future studies may investigate the long-term quality-of-life status among patients from different socioeconomic quintiles to clarify the population-based consequences of impaired or delayed access to care during pandemic settings. Moreover, it may be interesting to examine how a patient's location of residence impacts HCU and costs to expand our knowledge of the spatial impacts of access-to-care. These insights help to build more resilient health systems and facilitate the implementation of more equitable policies.

## Data Availability

The raw data supporting the conclusions of this article will be made available by the authors, without undue reservation.
